# Pre-hospital endotracheal intubation in severe traumatic brain injury: ventilation targets and mortality—a retrospective analysis of 308 patients

**DOI:** 10.1186/s13049-023-01115-8

**Published:** 2023-09-12

**Authors:** Jürgen Knapp, Pascal Doppmann, Markus Huber, Lorenz Meuli, Roland Albrecht, Stephen Sollid, Urs Pietsch

**Affiliations:** 1 Department of Anaesthesiology and Pain Medicine, Bern University Hospital, Inselspital, University of Bern, 3010 Bern, Switzerland; 2 Swiss Air-Rescue (Rega), Zurich, Switzerland; 3 Department of Anaesthesiology and Pain Medicine, University Hospital Basel, Basel, Switzerland; 4 Faculty of Medicine, University of Bern, Bern, Switzerland; 5 Department of Vascular Surgery, University Hospital Zürich, Zurich, Switzerland; 6 Department of Emergency Medicine, Bern University Hospital, Inselspital, University of Bern, Bern, Switzerland; 7 Division of Prehospital Services, Oslo University Hospital, Oslo, Norway; 8 Department of Anaesthesiology and Intensive Care Medicine, Cantonal Hospital St. Gallen, St. Gallen, Switzerland; 9 Institute of Clinical Medicine, Faculty of Medicine, University of Oslo, Oslo, Norway

**Keywords:** Prehospital emergency medicine, HEMS, Traumatic brain injury, Tracheal intubation, Ventilation

## Abstract

**Background:**

Traumatic brain injury (TBI) remains one of the main causes of mortality and long-term disability worldwide. Maintaining physiology of brain tissue to the greatest extent possible through optimal management of blood pressure, airway, ventilation, and oxygenation, improves patient outcome. We studied the quality of prehospital care in severe TBI patients by analyzing adherence to recommended target ranges for ventilation and blood pressure, prehospital time expenditure, and their effect on mortality, as well as quality of prehospital ventilation assessed by arterial partial pressure of CO_2_ (PaCO_2_) at hospital admission.

**Methods:**

This is a retrospective cohort study of all TBI patients requiring tracheal intubation on scene who were transported to one of two major level 1 trauma centers in Switzerland between January 2014 and December 2019 by Swiss Air Rescue (Rega). We assessed systolic blood pressure (SBP), end-tidal partial pressure of CO_2_ (PetCO_2_), and PaCO_2_ at hospital admission as well as prehospital and on-scene time. Quality markers of prehospital care (PetCO_2_, SBP, prehospital times) and prehospital ventilation (PaCO_2_) are presented as descriptive analysis. Effect on mortality was calculated by multivariable regression analysis and a logistic general additive model.

**Results:**

Of 557 patients after exclusions, 308 were analyzed. Adherence to blood pressure recommendations was 89%. According to PetCO_2,_ 45% were normoventilated, and 29% had a SBP ≥ 90 mm Hg *and* were normoventilated. Due to the poor correlation between PaCO_2_ and PetCO_2_, only 33% were normocapnic at hospital admission. Normocapnia at hospital admission was strongly associated with reduced probability of mortality. Prehospital and on-scene times had no impact on mortality.

**Conclusions:**

PaCO_2_ at hospital admission is strongly associated with mortality risk, but normocapnia is achieved only in a minority of patients. Therefore, the time required for placement of an arterial cannula and prehospital blood gas analysis may be warranted in severe TBI patients requiring on-scene tracheal intubation.

**Supplementary Information:**

The online version contains supplementary material available at 10.1186/s13049-023-01115-8.

## Introduction

Traumatic brain injury (TBI) remains one of the main causes of mortality and long-term disability, affecting 50 million people worldwide per year and costing € 325 billion [1, 2]. In Europe, crude incidence rates of TBI range from 47 to 694 per 100,000 population per year, with mortality rates ranging from 9 to 28 per 100,000 population per year [3].

TBI is caused by an initial traumatic insult to the brain, which disrupts normal physiology of the tissues involved. These non-physiological states may then lead to secondary damage [1, 2, 4–7]. Hypotension, inadequate ventilation, and hypoxemia are among the non-physiological states that increase mortality after severe TBI [6, 8–10]. Early tracheal intubation and controlled ventilation in the prehospital setting can improve outcomes of patients with severe TBI [11–13].

Guidelines on resuscitation of TBI patients recommend a partial pressure of end-tidal carbon dioxide (PetCO_2_) between 4.4 and 5.4 kPa in intubated patients (“normoventilation”) and maintenance of systolic blood pressure (SBP) ≥ 90 mm Hg (“normotension”), despite weak evidence and controversy surrounding this concept of a unique threshold for hypotension in severe TBI [14–17]. While optimizing brain tissue physiology seems to reduce mortality, the time required for treatment of trauma is also considered an important factor influencing outcome of trauma patients [18, 19]. Therefore, we studied the quality of prehospital care in severe TBI patients by analyzing adherence to recommended target ranges for ventilation and blood pressure, prehospital time expenditure, and their effect on mortality.

## Methods

We reviewed data from all patients in the Swiss Air Ambulance (Rega) mission archives who were suspected to have severe TBI in the prehospital setting, requiring tracheal intubation on scene and were transported to one of two level 1 trauma centers (Cantonal Hospital St. Gallen and Bern University Hospital, Switzerland) between January 2014 and December 2019. Prehospital management was performed according to current treatment protocols used by the helicopter emergency medical service (HEMS) of Rega. These include rapid sequence induction and tracheal intubation at a Glasgow Coma Scale (GCS) Score ≤ 8. Capnography was used to guide ventilation with a target PetCO_2_ between 4.4 and 5.7 kPa (33–43 mm Hg). Whether the patients were ventilated manually using a bag valve mask or mechanically could not be ascertained from the mission reports. Target SBP of ≥ 90 mm Hg was achieved—if necessary—by intravenous administration of crystalloid volume or catecholamine support. Capnography and non-invasive blood pressure monitoring were performed using a Propaq® Multifunction Monitor (ZOLL Medical Corporation, MA, USA).

Recorded data included patient characteristics, initial GCS score, first SBP at admission to the hospital, first PetCO_2_ at admission, arterial partial pressure of carbon dioxide (PaCO_2_) within the first 5 min of admission, injury severity score (ISS), and on-scene time, as well as total prehospital time (defined as interval between alert of the helicopter emergency medical service crew and hospital arrival). Mission information was transferred from paper logs into a spreadsheet (Microsoft Excel, Microsoft Corporation, Redmond, WA, USA). Patients were then matched to hospital records using date of birth and admission date and time.

### Statistical methods

In terms of summary statistics, categorical variables are presented with counts and percentages. Continuous variables are presented as median and interquartile range (IQR) or mean and standard deviation (STD) as appropriate. Unadjusted univariable group comparisons in Table 2 were based on the chi-square test (categorical variables), Student’s t-test (continuous, normally distributed) and unpaired two-samples Wilcoxon test (continuous, skewed).

Correlation between PetCO_2_ and PaCO_2_ was evaluated by the Pearson correlation coefficient. Univariable, non-linear associations of the outcome mortality with the covariates systolic blood pressure, PetCO_2_ and PaCO_2_ were graphically examined by a logistic general additive model. To analyze the effect of time expenditure in the prehospital setting, we performed a multivariable logistic regression to examine the association of a set of known predictors of outcome after severe TBI with mortality. The discrimination capacity of the logistic regression models was evaluated by means of the area under the receiver operating curve (AUROC). Model fit was evaluated by means of Nagelkerke's pseudo-R-squared. All computations were performed with R Version 4.0.2.

### Ethics

The study was conducted in accordance with ethical standards as laid down in the Declaration of Helsinki and all national guidelines. The local ethics committees of Bern and St. Gallen (EKOS) approved the study. Both granted permission to use patient data without individual informed consent according to the federal act on research involving human beings and the ordinance on human research with the exception of clinical trials. The permission also covered the use of patient data regarding HEMS operations (ethics committee Bern: BASEC nr. 2020-01737, study nr. 4690 and EKOS: BASEC nr. 2020-01737, 20/122).

## Results

**Fig. 1:**
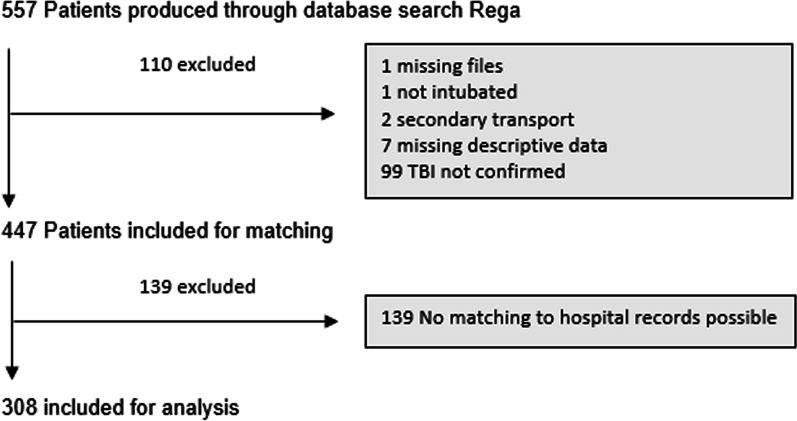
Patient flow chart. *TBI* traumatic brain injury

From January 2014 to December 2019 a total of 557 patients requiring tracheal intubation after suspected TBI were transported to the two participating trauma centers by Rega HEMS crews. A total of 110 patients were excluded due to a missing paper mission log (1), no documented intubation (1), being on an inter-hospital transfer (2), no description of the mission available (missing or illegible) (7) or no confirmation of intracranial injuries after diagnostic imaging (99). Of the remaining 447 patients, 139 patients could not be matched to corresponding hospital records. The remaining 308 patients were included for analysis (Fig. 1).

**Fig. 2:**
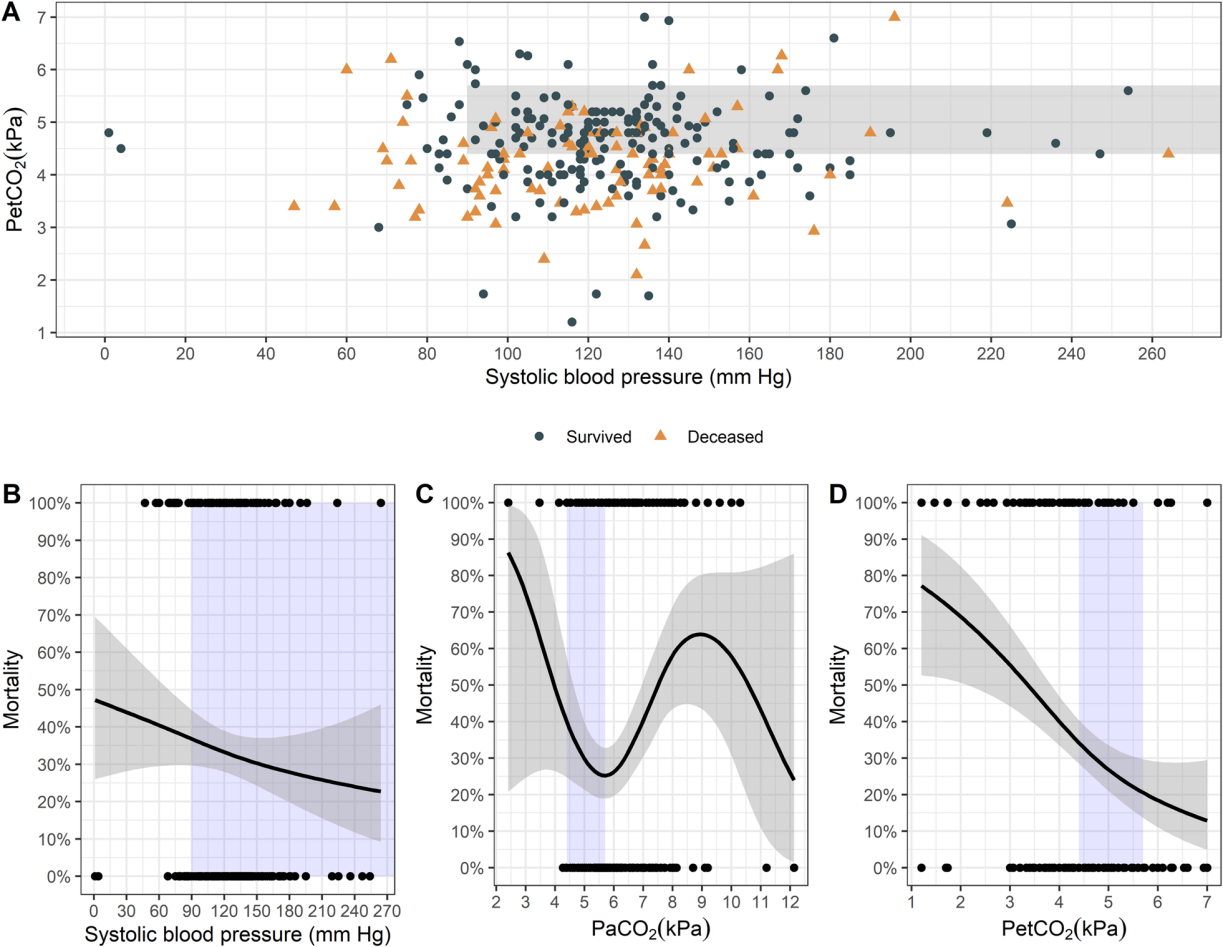
Scatter plot showing end-tidal partial pressure of CO_2_ (PetCO2) and systolic blood pressure at arrival in the resuscitation room for surviving and deceased patients, shaded area: normoventilation and systolic blood pressure ≥ 90 mm Hg (**A**). Probability of mortality depending on systolic blood pressure (**B**), arterial partial pressure of CO_2_ (PaCO_2_) (**C**) and PetCO_2_ (**D**) at arrival in the resuscitation room. Blue shaded area: Recommended treatment target. Grey shaded are: 95% confidence interval

Patient characteristics are shown in Table 1. The majority of TBI were sustained in road traffic accidents (45%), with accidents at home being the second most common cause (31%). Sporting accidents (13%) and accidents at work (8%) were lower in proportion, and classification of mechanism of injury was not available for eight patients (3%). The mortality rate was 36% (111/308). The median age of this cohort of patients was 52 years (IQR 27–71), and patients who survived were significantly younger. Median SBP in the resuscitation room was 122 mm Hg (IQR 104–138 mm Hg), with surviving patients having a higher median systolic blood pressure than those who died (unadjusted *p* = 0.045). SBP at handoff was ≥ 90 mm Hg in 89% of all patients. Vasopressors were administered to 24% of patients. Normoventilation according to the measured PetCO_2_ at arrival in the resuscitation room was achieved in 45% of the patients, and 29% were both normoventilated and had a systolic blood pressure ≥ 90 mm Hg (Fig. 2A).

**Table 1:** Baseline characteristics of the study population (n = 308)

	All	Survived	Died	*P*	N
	N = 308	N = 197	N = 111		
Age (years)	52 [27;71]	44 [23;64]	64 [37;79]	< 0.001	308
Sex				0.817	308
Male	234 (76%)	151 (77%)	83 (75%)		
Female	74 (24%)	46 (23%)	28 (25%)		
Glasgow Coma Scale (GCS)				< 0.001	304
4–8	154 (51%)	107 (55%)	47 (43%)		
9–15	46 (15%)	41 (21%)	5 (5%)		
3	104 (34%)	48 (24%)	56 (52%)		
Systolic blood pressure in resuscitation room (mm Hg)	122 [104;138]	124 [108;139]	119 [96;136]	0.045	293
PetCO_2_ in resuscitation room (kPa)	4.50 [3.87;4.93]	4.67 [4.10;5.07]	4.13 [3.47;4.70]	< 0.001	291
PaCO_2_ in resuscitation room (kPa)	5.99 [5.47;6.79]	5.87 [5.46;6.53]	6.40 [5.47;7.33]	0.019	270
Injury severity score	29 [20;38]	25 [17;34]	32 [25;41]	< 0.001	301
Vasopressor use				0.132	284
No	215 (76%)	145 (79%)	70 (70%)		
Yes	69 (24%)	39 (21%)	30 (30%)		
On-scene time (min)	30.0 [24.0;37.0]	30.5 [24.0;38.0]	29.0 [24.0;36.0]	0.392	307
Pre-hospital time (min)	59.0 [51.0;68.0]	60.0 [52.0;69.0]	59.0 [50.0;67.0]	0.223	305

PetCO_2_: end-tidal partial pressure of CO_2_. PaCO_2_: arterial partial pressure of CO_2_

**Fig. 3:**
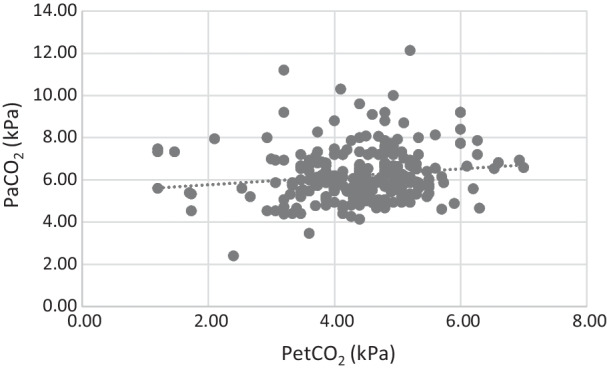
Correlation analysis of arterial partial pressure of CO_2_ (PaCO_2_) and end-tidal partial pressure of CO_2_ (PetCO_2_). Dotted line: regression line

As measured in the first arterial blood gas analysis in the emergency department, 33% of the patients had normocapnia at hospital admission. In surviving patients, PetCO_2_ was significantly higher (*p* < 0.001) and PaCO_2_ significantly lower (*p* = 0.019) compared to deceased patients. Correlation between PetCO_2_ and PaCO_2_ was weak with an r = 0.14 (Fig. 3, *p* = 0.02), and the mean difference was 1.81 kPa (SD 1.46 kPa). No difference in on-scene or total prehospital time was seen between surviving and deceased patients.

Factors significantly associated with in-hospital mortality in our multivariable regression analysis (Table 2**)** were greater age (OR 1.04, CI 1.03–1.06, *p* < 0.001), lower GCS (GCS 3 compared to GCS 4–8 OR 5.17, CI 2.31–12.3, *p* < 0.001), and higher ISS (OR 1.03, CI 1.00–1.06, *p* < 0.001). SBP at handover in the resuscitation room did not show any significant association with mortality (OR 1.0, CI 0.99–1.01, *p* = 0.5). Neither did on-scene time (OR 1.04, CI 0.99–1.09, *p* = 0.2) or total prehospital time (OR 0.97, CI 0.94–1.00, *p* = 0.079). These results are robust under multiple imputations that were performed as a sensitivity analysis (Additional file 1).

**Table 2:** Univariable and multivariable regression analysis of mortality

Characteristic	Univariable				Multivariable [N = 218]		
	N	OR	95% CI	*p*	OR	95% CI	*p*
Age (per year)	308	1.02	1.01, 1.03	< 0.001	1.04	1.03, 1.06	< 0.001
Sex	308						
Male		–	–		–	–	
Female		1.11	0.64, 1.89	0.7	0.66	0.28, 1.52	0.3
Glasgow Coma Scale (GCS)	304						
4–8		–	–		–	–	
9–15		0.28	0.09, 0.69	0.011	0.52	0.13, 1.71	0.3
3		2.66	1.59, 4.47	< 0.001	5.17	2.31, 12.3	< 0.001
Systolic blood pressure in resuscitation room (per mm Hg)	293	0.99	0.99, 1.00	0.10	1.00	0.99, 1.01	0.5
PetCO_2_ in resuscitation room (per kPa)	291	0.54	0.40, 0.71	< 0.001	0.55	0.35, 0.87	0.010
Injury Severity Score (per point)	301	1.04	1.02, 1.06	< 0.001	1.03	1.00, 1.06	0.037
Catecholamine	284						
No		–	–		–	–	
Yes		1.59	0.91, 2.77	0.10	0.83	0.36, 1.85	0.6
On-scene time (per min)	307	0.99	0.97, 1.01	0.4	1.04	0.99, 1.09	0.2
Pre-hospital time (per min)	305	0.99	0.97, 1.00	0.15	0.97	0.94, 1.00	0.079

PetCO_2_: end-tidal partial pressure of CO_2_, OR: odds ratio, CI: confidence interval

## Discussion

The pre-hospital phase is a very critical period for patients with severe TBI. Outcome after severe TBI is influenced by rapid interventions and early stabilization of ventilation and circulation, which together can prevent secondary brain damage [11, 12, 20, 21].

In our cohort upon hospital admission, recommended values for ventilation *and* blood pressure management according to the guidelines were achieved in only 29% of the patients after pre-hospital tracheal intubation. More importantly, however, we showed that PetCO_2_ does not reliably reflect PaCO_2_. In our patients, this led to 67% of the patients not being ventilated appropriately despite their PetCO_2_ being within the recommended range on hospital admission. The majority of these patients were hypercapnic (64%), whereas hypocapnia only occurred in 3% of cases.

The physiological causes of a gap between PaCO_2_ and PetCO_2_ are dead space ventilation and ventilation/perfusion mismatch. A gap of 0.4–0.7 kPa (3–5 mm Hg) is considered physiologic. In trauma patients, however, this gap can be markedly increased by hypovolemia and reduced cardiac output (due to hemorrhage, e.g.) as well as severe thoracic injuries. The proportion of normocapnic patients at hospital arrival in our study was even worse than in a study conducted *before* the availability of prehospital capnography, at a time in which ventilation was simply guided by estimated body weight [22]. This poor correlation is also clinically relevant when ventilation is performed in the pre-hospital setting with a self-inflating bag (e.g., during on-scene treatment and transfer from helipad to the emergency department) is typically titrated to PetCO_2_ and not with a ventilator with spirometry analysis. Hypocapnia may aggravate cerebral ischemia, and hypercapnia can increase intracranial pressure; both are known to negatively affect outcome of severe TBI patients [9].

Our findings underscore the tremendous importance of maintaining normocapnia already in the prehospital setting, before hospital admission (Fig. 2C). Hyperventilating patients (guided by PetCO_2_) seems to be independently associated with increased mortality (Fig. 2B, Table 2). This is in good accordance with and a potential explanation for the findings of a previous study by our group showing that the size of the CO_2_-gap between end-tidal and arterial measurements is significantly associated with mortality [23]. However, these results should not question the reliability of PetCO_2_ measurements altogether. Clinicians should consider the physiological causes of the CO_2_-gap and its potential increase in patients with hemodynamic instability or severe chest trauma. According to the results of another study, aiming for a PetCO_2_ of 3.3–4.0 kPa (25–30 mm Hg) in these patients could significantly increase the proportion of normocapnic patients upon arrival at the hospital [24]. Of course, in times of widespread availability of ultrasound and physicians that are well trained in prehospital emergency medicine, arterial blood gas analysis would be ideal and feasible.

The negative influence of hypotension on outcome in patients suffering from severe TBI has been proven in numerous studies, and recommendations for blood pressure management are integrated in all guidelines on emergency management of TBI [4, 8, 10, 14, 15, 25, 26]. Rega’s standard operating procedure for severe TBI patients uses a hypotension threshold of 90 mm Hg. Median SBP upon arrival at the trauma centers in our study was 122 mm Hg, and 89% of all patients had a SBP of ≥ 90 mm Hg, indicating good adherence to treatment recommendations for blood pressure.

Juelsgaard et al. analyzed adherence to company treatment recommendations for a Danish HEMS organization showing a SBP ≥ 90 mm Hg in 91% of TBI cases analyzed [4]. Further literature and guidelines recommend targeting higher SBP values between ≥ 100 mm Hg and ≥ 120 mm Hg [5, 27–30]. A SBP ≥ 120 mm Hg was achieved in only 55% of patients by Juelsgaard and colleagues, and 51% in another Danish study, exactly corresponding to our rate of 55% [4, 31]. However, the concept of a hypotension threshold as defined in international guidelines has been questioned recently, as these numbers are based on studies that mainly focused on dichotomous comparison of low and high blood pressure groups with arbitrary cutoffs [16].

New data show that mortality after TBI does not drop off at the proposed cut-off values for SBP. Spaite et al. found a rather linear association of lowest prehospital SBP and mortality, without any detectable threshold between 40 and 119 mm Hg [17]. Our unadjusted data supports this finding (Fig. 2B). After adjusting for several other factors, we found no significant association between blood pressure at hospital admission and mortality. One explanation for this could be that we only evaluated the blood pressure value at a certain point in time; however, the episodes of hypotension during the entire phase of emergency medical care are probably much more decisive for the outcome [4, 8]. We only evaluated the blood pressure values at hospital admission that were automatically recorded and documented in the resuscitation rooms. Blood pressure values during the prehospital phase were documented manually using paper mission logs which are known to be unreliable in terms of data quality. Additionally, more than 90% of patients had the recommended SBP of ≥ 90 mm Hg at hospital admission. These factors might also explain the lack of association between blood pressure and mortality in the regression analysis of our study.

In our cohort, we saw no significant association between on-scene or prehospital time and mortality. This might seem counterintuitive at first glance, as longer duration of prehospital times prolongs the timespan before severe TBI patients have access to more comprehensive diagnostic and treatment options in a critical care setting [32]. This potential delay can be hypothesized to increase the likelihood of secondary brain damage, and a shorter time on the scene before diagnosis could help improve outcomes [33–35]. However, the concept of the shortest possible time to definitive treatment being the sole indicator of outcome has been called into question [36–39]. More than simply increasing the speed at which patients are brought to definitive care, it seems that the impact of HEMS on mortality in severe TBI patients is a multifactorial process involving pre-hospital interventions which slow the development of secondary brain damage before transport [40]. In severe TBI patients in particular, adherence to physiological treatment targets as well as direct transport to level 1 trauma centers may have led to better outcomes, and not necessarily short prehospital times [1, 13, 41, 42].

In a comparison of physician- and non-physician-based HEMS services, Pakkanen and colleagues showed higher rates of pre-hospital interventions and conversely lower rates of non-physiologic vital parameters at admission. They interpreted this as being at least partially responsible for the observed improvement in mortality in the physician-based HEMS group [33, 43]. This might also help explain improved outcomes despite there being no time benefit or in some cases even longer prehospital times in other studies on physician-staffed HEMS [43–45]. One must also consider that indicated invasive emergency procedures in the prehospital setting do not increase *total* resuscitation time (i.e., time to surgery or an intensive care unit), as these procedures must be made up in the resuscitation room anyway [46]. Moreover, even though patients required rapid sequence induction and tracheal intubation, this study showed on-scene times of only 30 min. Finally, the high density of trauma centers in Switzerland, with relatively short approaches and transport times compared to other countries (interval from alerting the HEMS crew to arrival at the hospital was < 68 min for the vast majority of our patients) could also explain the lack of association between prehospital time and mortality [47].

### Limitations

Our study has several limitations. First, it was conducted in a retrospective fashion, which affected availability of data in some cases. Second, paper logs were used for HEMS missions. It is reasonable to assume that there was significant variability in the quality of logged data in the setting of severely injured patients requiring tracheal intubation, which represents a high workload for the entire team. We used the first values recorded in-hospital for SBP and PetCO_2_, as these were automatically recorded every minute and more reliable compared to the prehospital values recorded manually. Therefore, we cannot make any statement about the course of these parameters during prehospital care. However, we used the most valid data available. In the future, electronic recording of vital signs during prehospital care (as now introduced by Rega) may allow studies that are more accurate. Third, data may be difficult to compare due to the countries and regions with a lower density of hospitals and trauma centers, as well as different geographical challenges in prehospital missions. Forth, 139 out of eligible 447 patients could not be matched to corresponding hospital records (31%). The most common reason was that the patient's personal details were not stored or not correctly stored in the electronic documentation system during resuscitation room care and subsequently the data from resuscitation room care is missing in the patient record. This was certainly by chance and is therefore unlikely to have biased our results. And finally, neurological outcome at discharge could not be analyzed as it was not readily available for a majority of patients.

## Conclusion

In conclusion, we saw adherence to guideline recommendations in patients with severe TBI requiring tracheal intubation on scene, which was good for blood pressure management, but only moderate on normoventilation. However, due to the poor correlation between PetCO_2_ and PaCO_2_, only a minority of patients were normocapnic on hospital admission. Our results, although limited by the univariate exploratory analysis in this study, suggest that striving for normocapnia already in the prehospital phase may reduce patient mortality. As on-scene time did not have a significant impact on in-hospital mortality, the time required for placement of an arterial cannula and prehospital blood gas analysis may be beneficial to severe TBI patients requiring prehospital tracheal intubation.

### Supplementary Information

**Additional file 1**. Sensitivity analysis of multivariable regression analysis using multiple imputations.

## Data Availability

The datasets generated and/or analysed during this study can be obtained from the corresponding author on reasonable request.
