# Supplementary material for: Pre-hospital endotracheal intubation in severe traumatic brain injury: ventilation targets and mortality—a retrospective analysis of 308 patients
Source: Scand J Trauma Resusc Emerg Med. 2023 Sep 12;31:46. doi: 10.1186/s13049-023-01115-8 (PMC10498564; doi:10.1186/s13049-023-01115-8)
Supplement: Supplementary file 1 — Additional file 1. Sensitivity analysis of multivariable regression analysis using multiple imputations. [file 13049_2023_1115_MOESM1_ESM.docx]

|  | **Univariable** | | | | **Multivariable [N=218]** | | | **Multivariable (multiple imputation) [N=308]** | | |
| --- | --- | --- | --- | --- | --- | --- | --- | --- | --- | --- |
| **Characteristic** | **N** | **OR^1^** | **95% CI^1^** | ***p*** | **OR^1^** | **95% CI^1^** | ***p*** | **OR^1^** | **95% CI^1^** | ***p*** |
| **Age** (per year) | 308 | 1.02 | 1.01, 1.03 | <0.001 | 1.04 | 1.03, 1.06 | <0.001 | 1.03 | 1.02, 1.04 | <0.001 |
| **Sex** | 308 |  |  |  |  |  |  |  |  |  |
| Male |  | — | — |  | — | — |  | — | — |  |
| Female |  | 1.11 | 0.64, 1.89 | 0.7 | 0.66 | 0.28, 1.52 | 0.3 | 0.70 | 0.35, 1.40 | 0.3 |
| **Glasgow Coma Scale** (GCS) | 304 |  |  |  |  |  |  |  |  |  |
| 4-8 |  | — | — |  | — | — |  | — | — |  |
| 9-15 |  | 0.28 | 0.09, 0.69 | 0.011 | 0.52 | 0.13, 1.71 | 0.3 | 0.32 | 0.11, 0.93 | 0.036 |
| 3 |  | 2.66 | 1.59, 4.47 | <0.001 | 5.17 | 2.31, 12.3 | <0.001 | 3.65 | 1.89, 7.05 | <0.001 |
| **Systolic blood pressure in resuscitation room** (per mm Hg) | 293 | 0.99 | 0.99, 1.00 | 0.10 | 1.00 | 0.99, 1.01 | 0.5 | 1.00 | 0.99, 1.00 | 0.3 |
| **PetCO_2_ in resuscitation room** (per kPa) | 291 | 0.54 | 0.40, 0.71 | <0.001 | 0.55 | 0.35, 0.87 | 0.010 | 0.49 | 0.34, 0.69 | <0.001 |
| **Injury Severity Score** (per point) | 301 | 1.04 | 1.02, 1.06 | <0.001 | 1.03 | 1.00, 1.06 | 0.037 | 1.04 | 1.02, 1.07 | <0.001 |
| **Catecholamine** | 284 |  |  |  |  |  |  |  |  |  |
| No |  | — | — |  | — | — |  | — | — |  |
| Yes |  | 1.59 | 0.91, 2.77 | 0.10 | 0.83 | 0.36, 1.85 | 0.6 | 0.86 | 0.41, 1.79 | 0.7 |
| **On-scene time** (per min) | 307 | 0.99 | 0.97, 1.01 | 0.4 | 1.04 | 0.99, 1.09 | 0.2 | 1.02 | 0.99, 1.06 | 0.2 |
| **Pre-hospital time** (per min) | 305 | 0.99 | 0.97, 1.00 | 0.15 | 0.97 | 0.94, 1.00 | 0.079 | 0.98 | 0.95, 1.00 | 0.080 |
| ^1^OR = Odds Ratio, CI = Confidence Interval | | | | | | | | | | |

Sensitivity Analysis of multivariable regression analysis using multiple imputations. PetCO_2_: end-tidal partial pressure of CO_2_.
